# Two Cases of Malpositioning of Internal Jugular Central Venous Catheters: A Review of Literature and Current Treatment Recommendations

**DOI:** 10.7759/cureus.59814

**Published:** 2024-05-07

**Authors:** Luis Rodriguez, Reshma Pydi, Paul Joon Koo Choi, Jigyasha Pradhan, Sania Thite, Savni Satoskar, Harsh R Parikh, Ajay Shah, Hanasoge Girishkumar

**Affiliations:** 1 Surgery, BronxCare Health System, New York, USA

**Keywords:** intraarterial placement of cvc, complications of cvc placement, cvc malposition management, arterial puncture of cvc, malposition of cvc

## Abstract

Percutaneous central venous catheterization, despite ultrasound guidance, is known to carry significant risks. While central venous catheters are widely used in clinical practice, they are also associated with various complications, including incorrect positioning during insertion. Arterial puncture is a well-recognized complication, and although unintended subclavian or carotid artery cannulation is rare, it can lead to serious consequences. We present two cases, in which a dual-lumen, non-tunneled temporary hemodialysis catheter was inadvertently inserted into the left common carotid artery and in the left innominate vein.

## Introduction

Central venous catheters (CVC) serve as important conduits for the delivery of hyperosmolar drugs, chemotherapy, blood products, and parenteral nutrition [1,2]. They are also utilized in central venous pressure measurements, pulmonary artery catheterization, cardiac pacing, renal replacement therapy, and as an alternative venous access route when peripheral access is unavailable [1,2]. Common insertion sites for central catheters include the internal jugular and subclavian veins, followed by the femoral veins and superficial veins of the upper extremities [2]. Among these options, the right internal jugular vein (IJV) is often preferred due to its direct trajectory into the right atrium and reportedly lower risk of stenosis and thrombosis [1].

The placement of central venous catheters (CVC) necessitates awareness of potential complications, which typically fall into three categories: mechanical, infectious, and thrombotic [2]. Among the mechanical complications encountered during insertion, the three most common are arterial puncture, hematoma formation, and pneumothorax development [2]. Other notable complications include hydrothorax, hemothorax, chylothorax, cardiac tamponade, mediastinal hemorrhage, and mispositioning of the catheter tip [2]. Arterial puncture is a well-documented complication that occurs in 6-10 % of all IJV cannulation attempts and stands out as the most frequently encountered mechanical complication [3]. We present two cases of malpositioned CVCs to elucidate the current recommendations and guidelines on diagnosing, managing, and treating such patients.

## Case presentation

Case 1 

A 54-year-old female with a medical history of hypertension, diabetes, brain tumor, and stroke was brought to the emergency room with altered mental status and septic shock. On presentation, her creatinine was 2.8 mg/dl, which subsequently rose to 8.3 mg/dl on a repeat blood test. She developed multiorgan failure, leading to acute kidney injury (AKI) necessitating hemodialysis. Given the emergent need for dialysis, the vascular surgery team was consulted for the placement of a non-tunneled dialysis catheter. 

**Figure 1:**
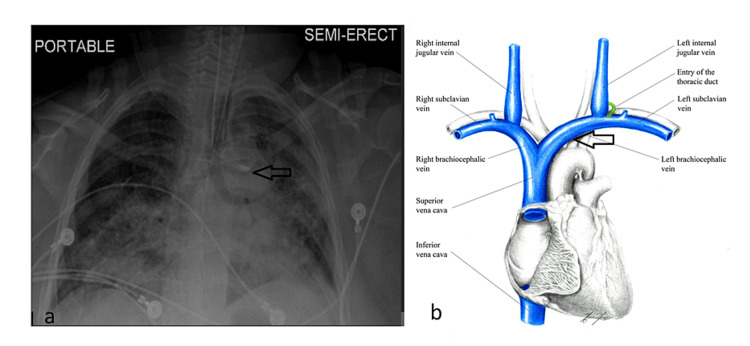
a. A right-sided central venous catheter takes an erroneous turn and appears to terminate in the left innominate vein (black arrow); b. Schematic diagram depicting the jugular and carotid anatomy, the black arrow indicates the location of the malpositioned catheter tip Source: Major Veins of the Heart 2 by ShannaBear on DeviantArt [4]

The patient was positioned in the Trendelenburg position, and the decision was made to use the right internal jugular vein (IJV) for central venous catheter (CVC) placement. Following skin preparation, landmark identification, and local anesthetic injection, the introducer needle was inserted under real-time ultrasound guidance. Blood was aspirated, and the needle was held in place to prevent migration. A guidewire was then advanced through the introducer needle, followed by the insertion of a dilator and the catheter over the wire without any resistance. All catheter ports spontaneously filled with blood. However, there were appreciable pulsations; therefore, a blood sample was drawn for gas analysis, revealing the intra-arterial placement of the catheter (pH: 7.34, pO2: 86.2 mmHg, pCO2: 34.4 mmHg, SpO2: 96%), which was further confirmed by the pressure waveform obtained from the catheter transducer. Although the chest X-ray (Figure 1) displayed the catheter tip in the left innominate vein (the radiologist reported an anomaly due to the left common carotid artery's anomalous origin from the right innominate artery), the decision was made to proceed to the operating room for further intervention.

**Figure 2:**
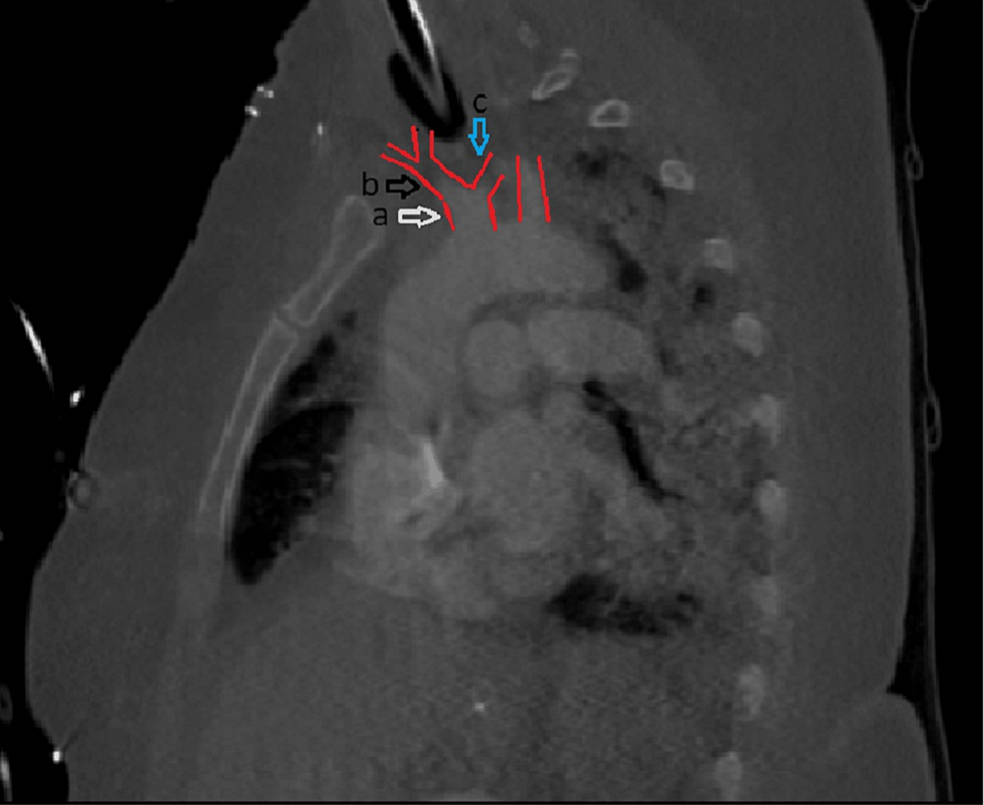
a (white arrow): The outlines of the common origin of the innominate artery and the left common carotid artery are delineated in red; b (black arrow): The innominate artery; c (blue arrow): The left common carotid artery

The patient was prepped for neck exploration and possible median sternotomy. During the exploration, it was noted that the catheter took a through-and-through passage from the right internal jugular vein into the innominate artery, continuing further into the left common carotid artery. The right subclavian artery, right common carotid artery, and innominate artery were dissected and secured with vessel loops, along with the left common carotid artery. Gentle traction on the vessel loops occluded arterial flow, allowing for the safe removal of the catheter. The puncture site in the innominate artery was carefully closed with interrupted 5-0 Prolene sutures, ensuring hemostasis upon release of the vessel loops. Repair of the internal jugular vein laceration was accomplished with a continuous 5-0 Prolene suture technique. Postoperatively, a CT angiogram of the neck and chest was obtained, which revealed an anomalous origin of the left common carotid artery from the innominate artery (Figure 2). The patient recovered without complications during the postoperative period.

Case 2 

A 58-year-old male with a non-functioning right radiocephalic arterial-venous fistula (AVF) and associated central venous stenosis presented to the emergency room with right upper extremity swelling. Following consultation with interventional radiology, the patient underwent a fistulogram and angioplasty of the AVF and subclavian vein. However, post-procedure, the primary team encountered difficulties in cannulating the AVF for hemodialysis. Consequently, the vascular surgery team was consulted for the emergent placement of a non-tunneled dialysis catheter.

**Figure 3:**
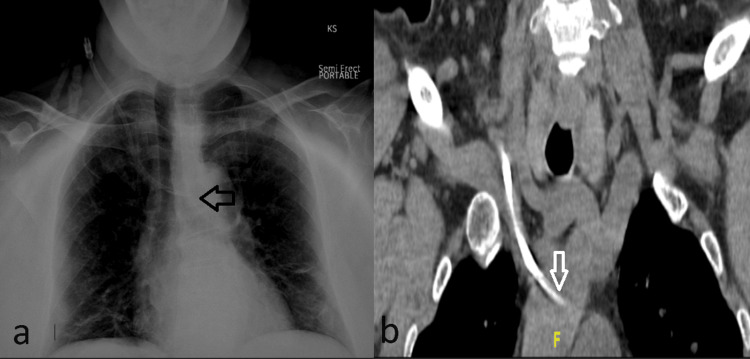
a. Right internal jugular central venous catheter in situ with its tip projecting into the region of the mid-left innominate vein (black arrow) b. The right jugular catheter tip was seen within the innominate vein (white arrow).

Under ultrasound guidance and standard aseptic technique, a non-tunneled hemodialysis catheter was inserted into the right internal jugular vein. However, a subsequent chest X-ray revealed an unusual course of the catheter, indicating a deviated course toward the mediastinum (Figure 3). Further evaluation via computed tomography imaging (Figure 3) confirmed the catheter tip's location within the left innominate vein. Duplex evaluation of the right neck demonstrated a patent right internal jugular vein with compressible and spontaneous phasic flow. The catheter tip was again observed to be situated in the innominate vein.

The patient was then prepared for surgery, positioned supine on the operating table, and the right neck and chest were prepped and draped in a standard sterile fashion. A guidewire was inserted into the blue port of the existing catheter, and the catheter was retracted to the level of the superior vena cava (SVC) under fluoroscopic guidance. Subsequently, a guidewire was inserted, and its tip was positioned in the inferior vena cava (IVC). The catheter was then advanced over the guidewire, with its tip placed at the junction of the SVC and the right atrium. Blood was aspirated from both ports, and the completion of fluoroscopy confirmed the patent left innominate vein and distal SVC, confirming the correct placement of the right internal jugular catheter with its tip within the distal SVC. The patient had an uneventful postoperative course.

## Discussion

Central venous catheters (CVCs) serve multiple purposes, including the delivery of blood products, resuscitative fluids, parenteral nutrition, and chemotherapeutic drugs [1,2]. They are also used for monitoring central venous pressure, pulmonary artery catheterization, transvenous cardiac pacemaker placement, and assisting in hemodialysis [1,2]. Despite the widespread use, CVCs are associated with various complications, some of which arise from the initial insertion procedure [1,2]. The most common complications include hematoma (up to 26%), infection (up to 26%), and pneumothorax (up to 30%) [5]. Mispositioning of the CVC tip during insertion is estimated to occur in 7-15% of all cases [1,5,6]. While accidental carotid arterial puncture is a well-documented complication, occurring in 6-10% of internal jugular catheterizations, the use of ultrasound guidance has reduced this incidence to 1.1% [7]. Misplacement of the CVC tip outside the lumen of the superior vena cava (SVC) increases the risk of secondary complications, such as catheter wedging, vascular erosion and puncture, endothelial damage-induced venous thrombosis, catheter malfunction, and cranial retrograde injection, where the infusate is directed towards the head instead of the intended central circulation [1,5].

Appropriate methods to confirm the intravenous location of the sheath after a puncture include but are not limited to the use of ultrasound, manometry, or pressure waveform analysis [8]. It is important to note that the color of the aspirated blood from the ports or the presence of pulsatile flow are not sensitive indicators to rule out misplacement [3]. Additionally, confirmation of the placement of the wire within the intravenous lumen and along the length of the vein using portable ultrasound is highly recommended prior to introducing a dilator.

**Figure 4:**
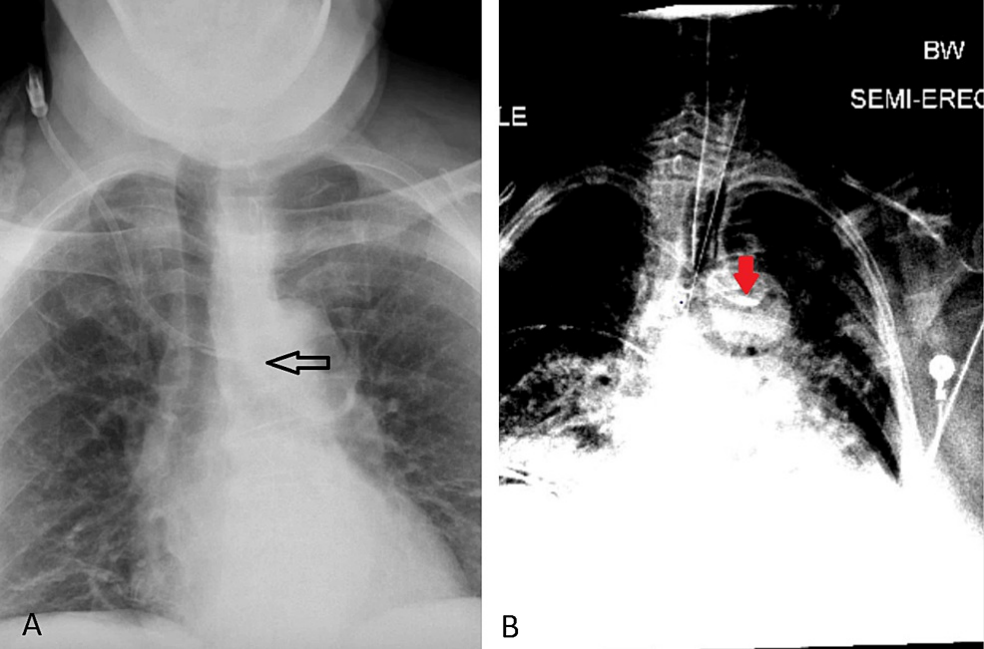
A: The catheter tip appears to be in the left innominate vein (black arrow); B: The catheter tip seemed to be in the innominate vein but was found to be in the left common carotid artery (red arrow)

If there is any uncertainty regarding whether the catheter or wire resides in the vein, confirmation is required using bedside portable ultrasound, transthoracic echocardiogram (TEE), continuous electrocardiogram (EKG), or fluoroscopic imaging [6,8]. The current literature indicates manometry is more sensitive at detecting arterial punctures not identified by blood flow and color compared to bedside ultrasound or pressure waveform analysis [8]. Observational studies indicate that TTE and continuous EKG are more effective in identifying proper catheter tip placement [8]. X-ray findings can be misleading [3]. For instance, Flavia Ramos Tristao et al. presented a case of a 48-year-old woman who underwent chemo port placement for breast cancer, the patient’s initial chest X-ray appeared to show a well-positioned catheter but when further computed tomography imaging was obtained, the catheter tip was found in the internal thoracic vein [2]. In our presented cases, both images indicated that the catheter tip was in the left innominate vein but in the first case, the tip was found in the left common carotid artery due to the aberrant anatomy of the patient’s bovine arch (Figure 4). Therefore, a chest X-ray must be followed by confirmation imaging if there is uncertainty about the correct placement of a central venous catheter.

In our first case, the decision to confirm the suspected intra-arterial placement of the catheter was expedited by observed filling of the lumens with pulsating blood and analysis of the pressure waveform. According to the current American Society of Anesthesiologists (ASA) guideline, if inadvertent cannulation of the artery is confirmed via imaging studies, the catheter should be left in place, and immediate consultation with general surgery, vascular surgery, or interventional radiology should be sought [8].

Treatment options for inadvertent arterial cannulation of a central venous catheter (CVC) include the use of percutaneous closure devices [9], temporary balloon tamponade with concurrent external manual pressure application [3,10], and open surgical exploration with or without vascular reconstruction [11]. Currently, there is no universal guideline to support the management of such patients, and treatment interventions are individualized based on various factors such as comorbidities, anticoagulation status, diameter of the CVC, and the site and timing of misplacement [3,8].

While open surgical exploration may be considered the ideal treatment, endovascular management has also shown effectiveness, with a success rate of 94.6% compared to 100% for open repair [3]. Open surgical methods are recommended when the injury is recognized more than four hours after cannulation or when there is no endovascular service available [12]. Endovascular repair offers advantages for patients with significant comorbidities and can avoid the need for general anesthesia. Additionally, endovascular techniques should be considered in cases of injury to the subclavian artery, which can be challenging to manually compress and access via an open surgical route [13].

## Conclusions

Bedside placement of central venous catheters without fluoroscopy can be challenging. Even under bedside ultrasound guidance, the rate of arterial puncture is greatly diminished but not eliminated. Confirming the position of the thin-wall needle or the guidewire in the vein using a portable ultrasound device before and after the induction of a dilator is crucial. An initial chest X-ray following placement can provide a clue to the catheter's position. However, appropriate methods to confirm the intravenous location of the sheath include but are not limited to the additional use of manometry, blood gas analysis, or pressure waveform analysis. If there is any uncertainty about the location of the catheter or guidewire, continuous EKG, fluoroscopic imaging, or TTE, which will identify the position of the tip of the catheter in the great vessels, is often used. The misplaced catheter should be sutured in place and prompt surgical or interventional radiology consultation must be sought. Treatment options span from the use of a percutaneous closure device, temporary balloon tamponade with external manual pressure, open surgical exploration with or without vascular reconstruction, to endovascular stent placement when feasible. Currently, there is no universal guideline for the management of misplaced CVC and treatments are highly individualized.
